# The Sensitivity of Moss-Associated Nitrogen Fixation towards Repeated Nitrogen Input

**DOI:** 10.1371/journal.pone.0146655

**Published:** 2016-01-05

**Authors:** Kathrin Rousk, Anders Michelsen

**Affiliations:** 1 Department of Biology, Terrestrial Ecology Section, University of Copenhagen, Universitetsparken 15, DK-2100, Copenhagen, Denmark; 2 Center for Permafrost (CENPERM), University of Copenhagen, Øster Voldgade 10, DK-1350, Copenhagen, Denmark; University of Saskatchewan, CANADA

## Abstract

Nitrogen (N_2_) fixation is a major source of available N in ecosystems that receive low amounts of atmospheric N deposition. In boreal forest and subarctic tundra, the feather moss *Hylocomium splendens* is colonized by N_2_ fixing cyanobacteria that could contribute fundamentally to increase the N pool in these ecosystems. However, N_2_ fixation in mosses is inhibited by N input. Although this has been shown previously, the ability of N_2_ fixation to grow less sensitive towards repeated, increased N inputs remains unknown. Here, we tested if N_2_ fixation in *H*. *splendens* can recover from increased N input depending on the N load (0, 5, 20, 80, 320 kg N ha^-1^ yr^-1^) after a period of N deprivation, and if sensitivity towards increased N input can decrease after repeated N additions. Nitrogen fixation in the moss was inhibited by the highest N addition, but was promoted by adding 5 kg N ha^-1^ yr^-1^, and increased in all treatments during a short period of N deprivation. The sensitivity of N_2_ fixation towards repeated N additions seem to decrease in the 20 and 80 kg N additions, but increased in the highest N addition (320 kg N ha^-1^ yr^-1^). Recovery of N in leachate samples increased with increasing N loads, suggesting low retention capabilities of mosses if N input is above 5 kg N ha^-1^ yr^-1^. Our results demonstrate that the sensitivity towards repeated N additions is likely to decrease if N input does not exceed a certain threshold.

## Introduction

Nitrogen fixation is a main input of new N for N limited habitats like subarctic tundra [1]. While deposition of reactive N (N_r_) in these areas is low (0–2 kg N ha^-1^ yr^-1^) [2], the predicted increase in N_r_ input to N-limited ecosystems seems inevitable e.g [3], and the effects on major ecosystem functions, like N_2_ fixation, have yet to be comprehensively assessed.

Feather mosses like *Hylocomium splendens* are ubiquitous in subarctic tundra, covering large fractions of the ground [4] and can contribute significantly to ecosystem productivity [5]. Moreover, *H*. *splendens* is colonized by several genera of N_2_-fixing cyanobacteria [6], contributing fundamentally to the N pool in pristine habitats [7]. Nevertheless, N_2_ fixation is an energy costly process and is inhibited when N availability and N_r_ deposition is high [8, 9, 10], which could limit ecosystem N input via the N_2_ fixation pathway. In temperate regions, atmospheric N deposition is commonly above 10 kg N ha^-1^ yr^-1^ [11], resulting in low or no detectable N_2_ fixation activity in mosses [6, 11]. Although average N deposition in the boreal biome is usually below 3 kg N ha^-1^ yr^-1^ [8], higher N deposition (>10 kg N ha^-1^ yr^-1^) can occur locally [12]. Several studies have shown that N_2_ fixation in the feather moss *Pleurozium schreberi* is inhibited already at very low N loads (3–12 kg N ha^-1^ yr^-1^, see e.g. 8, 10). And other studies have demonstrated a recovery from N stress upon removal of the stressor [13]. Yet, the potential ability of N_2_ fixation in mosses to acclimatize to high N loads, that is, to grow less sensitive to the stressor, has not been investigated so far. For instance, moss growth could be promoted by N input [14], which could result in increased photosynthesis. This in turn would lead to a higher demand for N that could be covered by the cyanobacterial associates if external N input ceases. Further, N_2_ fixation in the two dominant feather mosses in the boreal biome seems to respond differently to N deposition. Nitrogen fixation in *P*. *schreberi* seems to be less sensitive to increased N input than N_2_ fixation in *H*. *splendens* [10, 15], which was inhibited after long-term (15 years) N additions of 12.5 kg N ha^-1^ yr^-1^ [15]. Surprisingly, N_2_ fixation in *P*. *schreberi* was also found to be inhibited by only 3 kg N ha^-1^ yr^-1^ additions in a different field study by the same authors [8], indicating that pre-sampling conditions [16] and site characteristics like throughfall N [10] and horizontal N deposition [15, 17] might have fundamental effects on nitrogenase activity. On the other hand, in a laboratory set-up [13], the authors found no inhibition of N_2_ fixation in *P*. *schreberi* at additions of 10 kg N, contrasting the results reported by [8], and [10] showing moss associated N_2_ fixation to be inhibited by road-derived N deposition of 4 kg N ha^-1^ yr^-1^ in boreal forests. Thus, although N input can impede N_2_ fixation in mosses, the threshold of N input above which N_2_ fixation is inhibited is not well defined. Also, moss-growth might not be limited by N when colonized by N_2_ fixing associates [18], highlighting that the effects of N input on mosses and associated N_2_ fixers is multi facetted and warrants further assessments.

Given the dominance of mosses in N-limited ecosystems and their potential role as key players in the N cycle, a better understanding of the effects of increased N loads as well as the ability of N_2_ fixation to recover from this stressor is of great importance to elucidate the functioning of N-limited habitats like subarctic tundra. Herein, we report results from a N addition experiment with *H*. *splendens* collected from a birch forest in subarctic tundra, Northern Sweden, in which we added different amounts of N to the moss, followed by a period of N deprivation and concluded with another N addition period. Our aims were to assess 1) the response of moss-associated N_2_ fixation towards a range of N additions, and 2) the sensitivity towards the same N loads after a period of N deprivation.

## Material and Methods

### Sampling

*Hylocomium splendens* samples were collected from a subarctic birch forest (*Betula pubescens*) close to Abisko Research Station, Northern Sweden (68°20’ N, 18°49’ E) in May 2014. The long-term annual mean temperature in this area is 0.5°C, the average July temperature is 11°C, and the mean annual precipitation is 304 mm (30 years mean from 1971–2001, http://polar.se/en/abisko-naturvetenskapliga-station/). The dominant plant species at this site are *Betula pubescens*, which forms the canopy, and the low shrubs *Empetrum hermaphroditum*, *Vaccinium vitis-idaea*, *V*. *myrtillus*, *V*. *uliginosum*. The dominant moss species are *Hylocomium splendens* and *Pleurozium schreberi*, with 25–60% ground cover [19]. The samples were taken by cutting 21 x 21 x 10 cm intact, unfrozen blocks of *H*. *splendens* from the forest floor. The samples were placed in white PVC boxes with the same dimensions and kept moist under ambient light until their transport to Copenhagen. Upon arrival in the laboratory in Copenhagen, the samples were kept moist in the growth chamber at 10°C for 18 h light and 2°C for 6 h dark prior (8 weeks) and throughout the duration of the experiment, which was initiated in July 2014.

The location for the moss sampling is not privately-owned, outside the nearby nature reserve, and the collected moss is not an endangered or protected species but rather one of the most abundant moss species in boreal and subarctic forests in Sweden, and worldwide. Abisko Research Station has a general agreement with the Swedish authorities to conduct research in the state-owned land area outside protected areas.

Our research does not include any ethical issues.

### Nitrogen additions

Nitrogen was added as ammonium nitrate (NH_4_NO_3_) in five different application rates to the moss: 0, 5, 20, 80, 320 kg N ha^-1^ yr^-1^, with five replicate samples per N addition.

One moss sample consisted of 15 moss shoots of approximately 4 cm length in 50 ml centrifuge tubes with a diameter of 2.6 cm. Fifteen shoots of *H*. *splendens* correspond to an area of 14.6 ±1.8 cm^2^, estimated by measuring the length and width of 15 moss shoots using 50 x15 replicates, see also [20]. This area estimate was used to calculate the N application rates.

Five ml of double distilled H_2_O, for the control treatment, or 5 ml of the N solutions were added to the moss. We collected the leachates by perforating the 50 ml tubes at the bottom. The N solutions were added weekly over a period of four weeks to not damage the moss and colonizing cyanobacteria by e.g. decreasing the internal pH. This resulted in an addition of ¼ of the final amount per week (i.e. 0.18, 0.74, 2.94, 11.76 mg N week^-1^ for the 5, 20, 80 and 320 kg N ha^-1^ yr^-1^ doses, respectively) and the final rates of 5, 20, 80, 320 kg N ha^-1^ yr^-1^ were reached after 4 weeks. A stock solution of 560 mg NH_4_NO_3_ l^-1^, corresponding to 320 kg N ha^-1^ yr^-1^, was diluted to yield 5, 20, 80 kg N ha^-1^ yr^-1^ solutions. After the complete N loads were achieved (after week 4), the moss was rinsed with 5 ml sterile, N-free water once per week to induce N deprivation. As soon as we noticed an increase in N_2_ fixation, which was after only two weeks of N deprivation, we started to add ¼ of the final N amount weekly to reach the second complete N application dose after an additional four weeks, i.e. at week 10.

### Acetylene reduction assay

Nitrogenase activity as a measure of N_2_ fixation was assessed with the acetylene reduction assay (ARA) as in [21]. This assay is an adequate method to measure nitrogenase activity in feather mosses like *H*. *splendens* and *P*. *schreberi*, which are associated with N_2_ fixing cyanobacteria [22]. By contrast, in *Sphagnum* mosses, the ARA likely underestimates nitrogenase activity due to the inhibitive effect of acetylene on methanotrophic N_2_ fixation [23, 24], which is the dominant form of N_2_ fixation in *Sphagnum* mosses [18, 24].

Acetylene reduction was measured before the start of the experiment and immediately after each N addition, as well as after each rinsing with sterile, N-free water. For that, the 15 shoots of *H*. *splendens* were placed in 50 ml centrifuge tubes, sealed with a rubber septa (Suba Seal, Sigma) and 10% of the headspace was replaced with acetylene. The samples were incubated for 24 h, in 18 h light at 10°C and 6 hours dark at 2°C. Six ml of the headspace was transferred into 6 ml pre-evacuated, air-tight vials (Labco, Ceredigion, UK) and analyzed for ethylene production with a gas chromatograph (SRI 310C, FID, SIR Instruments, California). To test for natural production of ethylene by *H*. *splendens*, we also incubated moss samples without acetylene in the first AR measurements. No natural production of ethylene by *H*. *splendens* was detected.

### Analyses of leachate samples

Three ml of leachate were collected immediately after each N addition as well as after rinsing with sterile, N-free water. These leachate samples were analyzed for total dissolved N (TDN), nitrate (NO_3_^-^) and ammonium (NH_4_^+^) with a flow injection analyzer (5000 FIASTAR, Höganäs, Sweden).

### Total N content of moss

At the end of the experiment, moss samples were dried (24 h at 70°C), ground and analyzed for total N (TN) content using a LECO CN analyzer.

### Statistical analyses

Linear regression analyses were used to test for the effects of N addition on nitrogenase activity at four “key times” separately—before the N additions started, after the first complete N addition cycle, after the rinsing period and after the second complete N addition cycle. To test for differences in nitrogenase activity between the N additions and time of measurement throughout the entire experiment, we used repeated measures ANOVA with time as factor. To conform with the assumptions of ANOVA, the ethylene data was log transformed. We present the original data in the graphs to ease comparisons with previously published data. Linear regressions were used to test for the relationship between N dose, nitrogenase activity and TN content of mosses and the N concentrations of the leachate samples. Inorganic N in the leachates (NH_4_^+^ and NO_3_^-^) is reported as % of the added N. All analyses were performed in R 3.0.3. [25].

## Results

### Effects of N additions on nitrogenase activity

**Fig 1:**
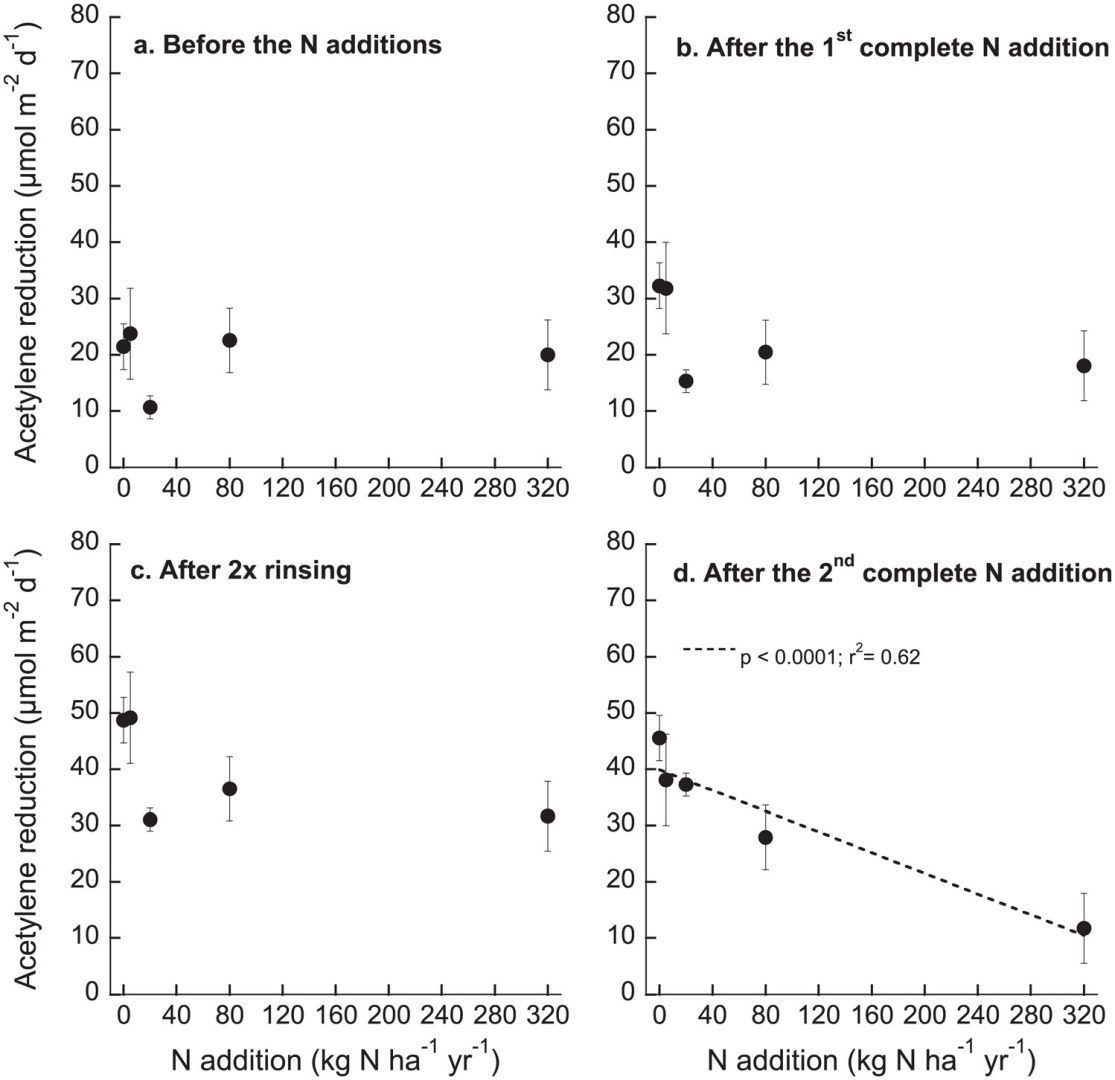
Acetylene reduction (μmol m^-2^ d^-1^) in moss samples exposed to five different N application rates (0, 5, 20, 80, 320 kg N ha^-1^ yr^-1^) at selected times during the experiment. **a** before the N additions started, **b** immediately after the 1^st^ full addition cycle (see also week 4 in Fig 2), **c** after 2 weeks of rinsing with N-free water (week 6 in Fig 2) and **d** immediately after the 2^nd^ full N addition cycle (week 10 in Fig 2). Given are means ± SE (n = 5). Regression lines are included if significant.

Results from the linear regression analyses showed that nitrogenase activity was similar in the mosses prior to the N additions (p = 0.95; r^2^ = 0.04; F_1,23_ = 0.004; Fig 1a). Also after the first complete N addition cycle, nitrogenase activity did not differ between the treatments (p = 0.23; r^2^ = 0.02; F_1,23_ = 1.49; df = 23; Fig 1b). This pattern was also found during the rinsing period (p = 0.17; r^2^ = 0.04; F_1,23_ = 1.97; Fig 1c), but nitrogenase activity decreased with increasing N load after the second complete N addition cycle (p < 0.0001; r^2^ = 0.62; F_1,23_ = 39.67; Fig 1d).

**Fig 2:**
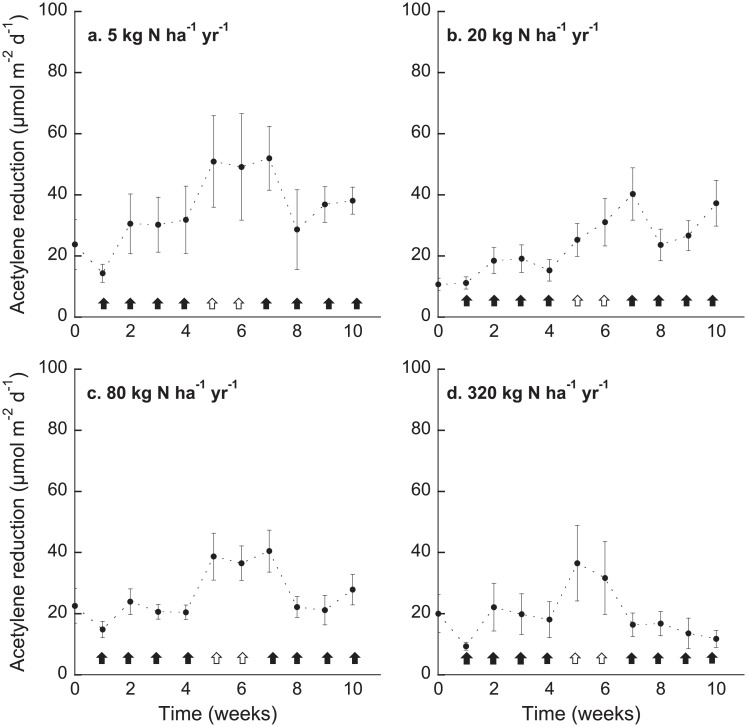
Mean acetylene reduction (μmol m^-2^ d^-1^) ±SE (n = 5) in moss samples over the course of the experiment (T0—T10). Four different N application rates (**a** 5, **b** 20, **c** 80, **d** 320 kg N ha^-1^ yr^-1^) were applied over a period of four weeks (1/4 of the final N amount per week), followed by a two-week deprivation phase, and concluded with another N addition cycle. The black arrows indicate the N addition periods, and the white arrows indicate the N deprivation (rinsing with N-free water) period. T0 is the acetylene reduction activity before the start of the N additions.

The results from the repeated measures ANOVA demonstrated that nitrogenase activity changed over the course of the experiment (p < 0.0001; F_1,8_ = 37.56) as well as in response to the N additions (p < 0.0001; F_1,1_ = 38.73; df = 1), and the effects of N addition on nitrogenase activity were dependent on time (p = 0.0002; F_1,9_ = 14.73; Fig 2). Nitrogenase activity in the 0 and 5 kg N additions were similar over the course of the experiment and were higher than activity in the 20 and 320 kg N ha^-1^ yr^-1^ additions (Fig 2). Mosses exposed to 80 kg N ha^-1^ yr^-1^ had higher activity over the course of the experiment than the mosses receiving 320 kg N ha^-1^ yr^-1^, and activity was, albeit numerically lower, not significantly different from activity in mosses receiving 0, 5 and 20 kg N ha^-1^ yr^-1^ (Fig 2).

Although nitrogenase activity in all N additions was inhibited after the first ¼ addition, activity tended to increase during the course of the first cycle of N additions (Fig 2 week 1–4). Rinsing the mosses two times with N-free water resulted in higher activity in all mosses than before the start of the N additions (week 0) as well as after the first ¼ addition (week 1) (Fig 2). Surprisingly, this was also true immediately after the first addition of N in the second cycle, except for the 320 kg N ha^-1^ yr^-1^ addition (week 7) (Fig 2). At this time, nitrogenase activity was also higher than during the first N addition cycle (Fig 2), which might indicate decreased sensitivity for N input. At the last time point (week 10), that is, after the second complete cycle of N additions, nitrogenase activity in the 5, 20 and 80 kg N additions was higher than after the first ¼ addition (week 1). However, these patterns did not hold for the highest N addition. After the second complete N addition cycle, nitrogenase activity in the highest N load (320 kg N ha^-1^ yr^-1^) decreased, and was lower than after the first cycle of N addition, likely indicating increased sensitivity to a high N load (Fig 2).

### N concentrations of leachates and total N content of the moss

**Fig 3:**
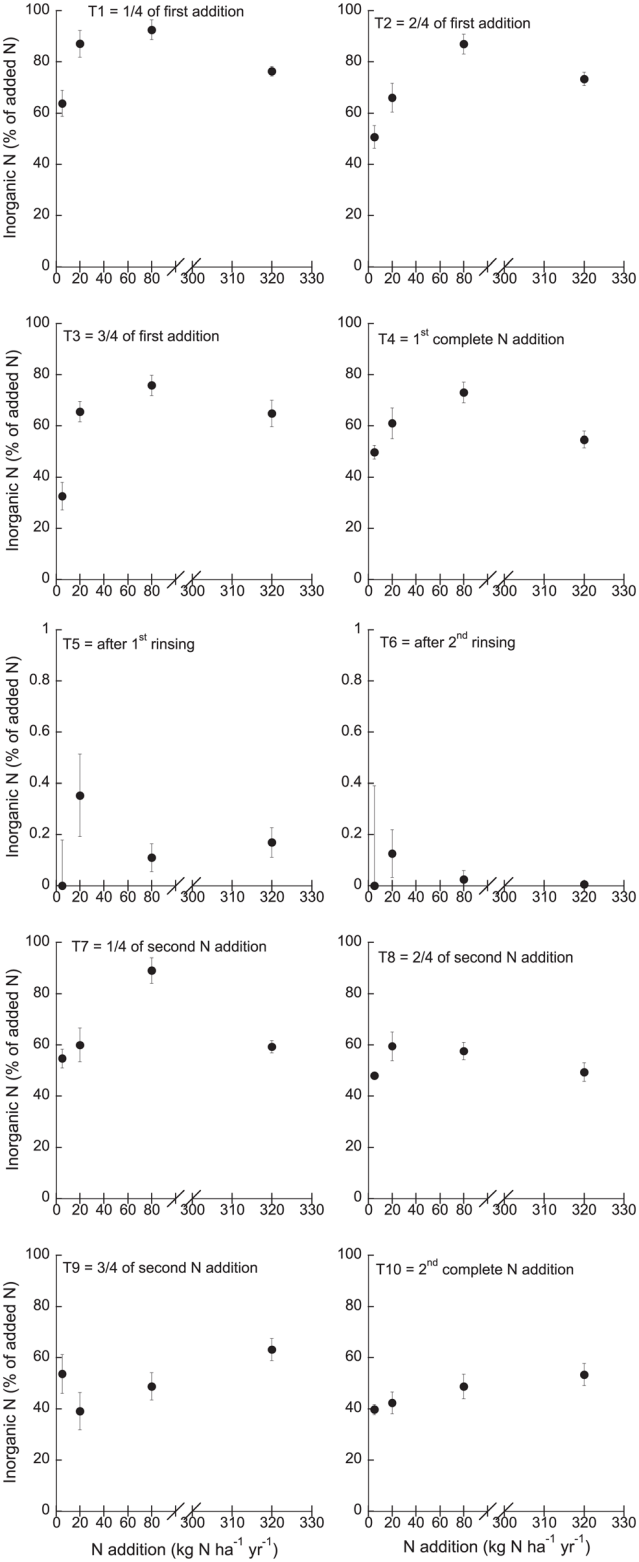
Inorganic N (NH_4_^+^ and NO_3_^-^) recovered in the leachates over the course of the experiment (% of added N). Given are means ±SE (n = 5). Please note the different y-scale for week 5 and 6 (N deprivation period).

Total dissolved N (TDN) in the leachate samples ranged between 1.05 mg l^-1^ and 121.4 mg l^-1^ and increased with increasing N addition (p < 0.0001; r^2^ = 0.69; F_1,248_ = 553.31), but remained similar during the experiment, except for the two weeks of N deprivation during which TDN was very low (S1 Fig). Similarly, NH_4_^+^ as well as NO_3_^-^ concentrations in the leachates were lowest during the two weeks of N deprivation but high otherwise (p_time_ = 0.006; t = -3.49 and p_time_ = 0.0004; t = -3.61 for NH_4_^+^ and NO_3_^-^, respectively; S1 Fig). Ammonium and NO_3_^-^ in the leachate increased with increasing N addition (p < 0.0001; r^2^ = 0.68; F_1,239_ = 518.72 and p < 0.0001; r^2^ = 0.68; F_1,248_ = 532.60 for NH_4_^+^ and NO_3_^-^, respectively). The recovery of the added N in the leachates was high and increased with increasing N addition (p = 0.01; t = 2.55), but decreased over time (p = 0.007; t = -2.73; overall model: p < 0.0001; r^2^ = 0.11; F_1,246_ = 11.01; Fig 3). The highest inorganic N concentrations in the leachates were recovered after the first ¼ addition (T1) (101.5%) and after the second ¼ addition (T7) (102.4%) in the 80 kg N ha^-1^ yr^-1^ treatment (Fig 3).

Acetylene reduction was negatively correlated with TDN (p < 0.0001; r^2^ = 0.20; F_1,246_ = 17.44), NO_3_^-^ (p < 0.0001; r^2^ = 0.17; F_1,246_ = 17.45), NH_4_^+^ (p < 0.0001; r^2^ = 0.18; F_1,237_ = 18.23) and the recovered inorganic N (p < 0.0001; r^2^ = 0.21; F_1,246_ = 23.34) in the leachate (S2 Fig).

**Fig 4:**
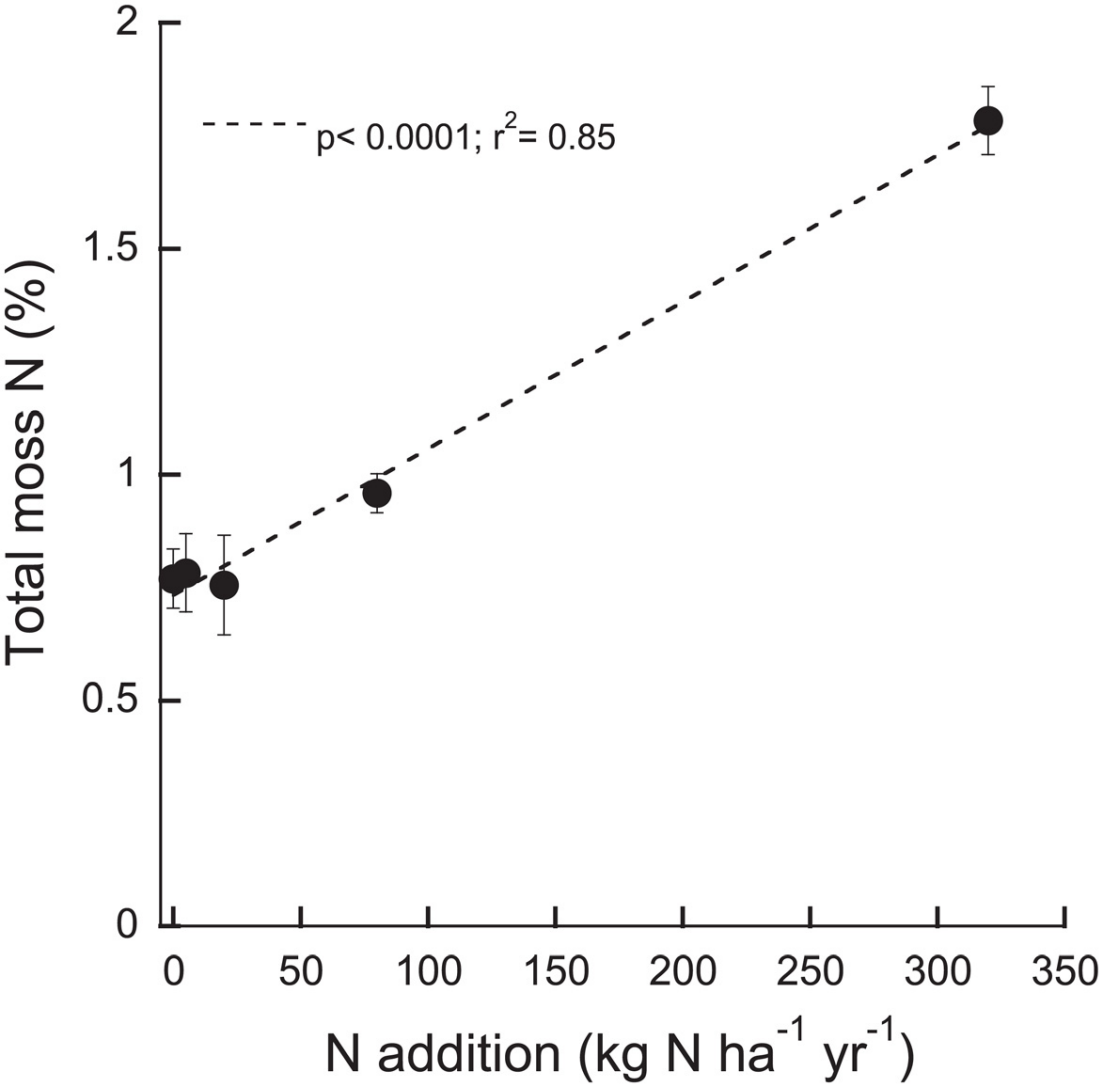
Total N (%) in the mosses exposed to different N loads at the end of the experiment. Given are means ±SE (n = 5).

At the end of the experiment, total N (TN) in moss tissue increased with increasing N load (p < 0.0001; r^2^ = 0.85; F_1,23_ = 134.61; Fig 4). Further, acetylene reduction was negatively related to TN in all treatments except for the control and the 5 kg N ha^-1^ yr^-1^ treatments (p < 0.0001; r^2^ = 0.65; F_1,21_ = 16.04).

## Discussion

The nitrogenase enzyme, which catalyzes the reduction of N_2_ to ammonia, is inhibited by its end product. Accordingly, moss-associated N_2_ fixation in boreal forests has been shown to be inhibited by N input [8, 9, 10]. Here, we tested the sensitivity of N_2_ fixation in one of the dominant moss species in the boreal biome, *H*. *splendens*, to different loads of N and its response to repeated short-term N input. We firstly aimed to evaluate the response of N_2_ fixation in *H*. *splendens* towards a range of N additions. Our N additions were high and short-term, and the effects on N_2_ fixation in this experimental set-up should be interpreted as a response to those high and short-term N loads, and not as absolute threshold values to estimate critical loads for ecosystems. Five kg N ha^-1^ yr^-1^ did not inhibit N_2_ fixation in *H*. *splendens*, but rather, seemed to enhance N_2_ fixation compared to the initial conditions (Fig 2, week 0). Surprisingly, the effect of 80 kg N ha^-1^ yr^-1^ additions on N_2_ fixation was not different from adding only 20 kg N ha^-1^ yr^-1^, which could be a consequence of the stepwise addition of N that prevented extreme exposures to N. Further, since we watered the mosses with the N solutions in an open-bottom set-up, the N might simply have been rinsed through too quickly to lead to a negative effect on N_2_ fixation. This is also indicated in the high recovery of inorganic N in the leachate samples (see below). Although our rinse-through system might have resulted in less exposure to N than if the mosses were soaked in a N rich solution, we could reduce N_2_ fixation with the highest N addition, and activity increased during the N deprivation period. Mosses do not possess roots, their connection to soil is limited to rhizoids, and they are dependent on nutrient uptake from the atmosphere. Furthermore, mosses cannot control water loss and easily leach nutrients to the soil [26]. Thus, the rinse-through system as well as the N deprivation period might be similar to the natural conditions the mosses are exposed to in nature. The lack of a strong and immediate inhibition of N_2_ fixation in the high N addition could also be a result of the type of N amendment used. While the applied solutions might have been quickly washed through the moss, solid N fertilizers used in other addition experiments likely lead to a slow and constant N release to moss and associated N_2_ fixers [8]. Further, laboratory incubations exclude any background N deposition rates including atmospheric N, throughfall N and horizontal N deposition, which could add more than 6 kg N ha^-1^ yr ^-1^ [10, 27], leading to a total N input to pristine ecosystems that can be above 10 kg N ha^-1^ yr^-1^ [13].

Increased N input to strictly N-limited ecosystems can change plant communities adapted to low N availability. For instance, adding 30 kg N ha^-1^ yr^-1^ over a period of 10 years in boreal forests in Northern Sweden lead to a decrease in *Sphagnum* moss cover, and an increase in vascular plant cover [28]. On the other hand, long-term (24 years) and high N input (50 kg N ha^-1^ yr^-1^) lead to an increase in moss cover and a decrease in vascular plant cover in arctic plant communities [29]. Similarly, more moss shoots of *H*. *splendens* grew in fertilized plots compared to control plots in subarctic tundra, although they changed morphology as they grew in the same core area, i.e. they were more abundant but “thinner” [14]. The moss in the higher N additions in our experiment also seemed “greener” than the moss in the control treatments, corresponding to their higher N content (Fig 4). These findings suggest that mosses could be N limited, despite associated cyanobacteria [30]. The mosses likely used the added N for growth and might not have been dependent on colonizing cyanobacteria as an N source. In addition, or alternatively, increased N within the moss tissue could have led to higher C fixation thus providing the colonizing cyanobacteria with the required energy to fix N_2_.

Our second aim was to assess if N_2_ fixation can recover from increased N load during a N deprivation phase, and if the response to a second cycle of N additions would change. Rinsing the moss only once with N-free water lead to an increase in N_2_ fixation in *H*. *splendens*, even after exposure to very high additions of 320 kg N ha^-1^yr^-1^ (Fig 2). Not surprisingly, the sensitivity of N_2_ fixation to repeated N additions depended on the amount of N added. While the lowest N addition (5 kg N ha^-1^ yr^-1^) did not seem to change the sensitivity of N_2_ fixation to another cycle of N additions, N_2_ fixation in mosses exposed to the higher N loads (20, 80 kg N ha^-1^ yr^-1^) did seem to become less sensitive to repeated N input. Nitrogenase activity was reduced upon repeated exposure to the highest N load only (320 kg N ha^-1^ yr^-1^), suggesting that N_2_ fixation grew more sensitive to the repeated exposure to high N. In the first round of N additions, the moss might have been more N-limited than after a full addition of N, thereby likely protecting the nitrogenase enzyme by assimilating the added N. During the N removal phase, N_2_ fixation increased again to cover the N demand of the moss [31]. Yet, after the second round of N additions, the moss was N saturated and could not assimilate additional N which could have led to inhibition of nitrogenase activity in the highest N addition (320 kg N ha^-1^yr^-1^). Hence, recovery of N_2_ fixation from increased N loads is possible [13], and N_2_ fixation can grow less sensitive to repeated additions of N, but only up to a threshold, which is likely higher than previously thought.

The increasing N concentrations with increasing N additions in the leachates suggest that the mosses cannot retain high loads of N, and that they release substantial amounts of N if supply exceeds demand. Rinsing the mosses with N-free water did not result in loss of N, indicating that during this period, N availability did not exceed the moss’ N demand, despite higher rates of N_2_ fixation. Either N_2_ fixation was not high enough to lead to excess N in the moss tissue, or the cyanobacteria simply retained the fixed N_2_ and did not transfer any N to the moss-host. This finding also implies that the previous high N additions did not lead to prolonged N excess and suggests quick recovery from high N load. The recovery of inorganic N in the leachates was lower after the second cycle of N additions than after the first additions (Fig 3). The moss might have stored the added N in the second cycle of additions. Taken together, these findings suggest that the moss-cyanobacteria associations are found along a continuum of interactions: the exchange of nutrients between the partners depends on the availability of N (and C) and the nutrient demand of the partners, see e.g. [32].

In a previous study, recovery of N_2_ fixation in the feather moss *P*. *schreberi* from long-term exposure to increased N loads was slow (>20 weeks) [13]. The authors induced N_2_ fixation in the moss collected from a high deposition area (12–15 kg N ha^-1^ yr^-1^; Newborough, UK). The slow recovery was due to very few numbers of colonizing cyanobacteria on the moss to begin with, as a result of historic high N deposition. In contrast, cyanobacterial colonization in our study was probably higher to start with compared to [13]. Further, the cyanobacteria likely remained on the moss leaves during the N additions, albeit inactive in a dormant state [33], but became active immediately upon removal of the stressor.

While our chosen N additions were high and short-term (4+4 weeks), ecosystems might be exposed to extreme N inputs for a short period during temporary high pollution output from industry. Yet, it is unlikely that mosses in the Subarctic will be exposed to N additions as high as 80 or 320 kg N ha^-1^ yr^-1^. We included these high additions as negative controls and to test the sensitivity of N_2_ fixation under controlled conditions. Nevertheless, we applied a range of N loads, from actually experienced (5 kg N ha^-1^ yr^-1^) to realistic loads at industry peaks (20 kg N ha^-1^ yr^-1^) and to extremely high N input (80 and 320 kg N ha^-1^ yr^-1^), which can help to increase our mechanistic understanding of the response of N_2_ fixation to acute doses of N. Hence, our study gives a first glimpse on how N_2_ fixation and N leaching processes in N-limited ecosystems might respond to extreme and short-term N loads.

Nitrogen deposition will increase with the expansion of agriculture and industry towards the North in the future, which might have dramatic consequences for ecosystems that are adapted to low N availability [28, 34]. However, moss associated N_2_ fixation can grow less sensitive to increased N input if a phase of N deprivation is provided and the N load does not exceed a certain threshold. Thus, increased N deposition to pristine ecosystems like subarctic tundra might not lead to decreased input via N_2_ fixation in mosses, if interspersed with periods of no or low N input.

## Supporting Information

S1 Data Tables of raw data of acetylene reduction, leachate as well as the total N data. (ZIP)

S1 Fig Total dissolved N (TDN), NO_3_^-^ -N and NH_4_^+^-N (mg/l) in moss leachates over the course of the experiment. Given are means ±SE (n = 5). Error bars are sometimes smaller than the symbols.
(DOCX)

S2 Fig Acetylene reduction (μmol m^-2^ d^-1^) in relation to total dissolved N (mg l^-1^), nitrate-N (mg l^-1^), ammonium-N (mg l^-1^) and recovered inorganic N (%) in the leachate samples. Shown are all data points measured throughout the experiment across all N addition treatments.
(DOCX)
