# Supplementary material for: The Sensitivity of Moss-Associated Nitrogen Fixation towards Repeated Nitrogen Input
Source: PLoS One. 2016 Jan 5;11(1):e0146655. doi: 10.1371/journal.pone.0146655 (PMC4712137; doi:10.1371/journal.pone.0146655)
Supplement: S1 Fig — Given are means ±SE (n = 5). Error bars are sometimes smaller than the symbols. (DOCX) [file pone.0146655.s002.docx]

**Supporting Information**

**Manuscript title: The sensitivity of moss-associated nitrogen fixation towards repeated nitrogen input**

**Authors:** Kathrin Rousk^1,2,*^, Anders Michelsen^1,2^

^1^Department of Biology, Terrestrial Ecology Section, University of Copenhagen, Universitetsparken 15, DK-2100, Copenhagen, Denmark. ^2^Center for Permafrost (CENPERM), University of Copenhagen, Øster Voldgade 10, DK-1350 Copenhagen, Denmark. *Corresponding author: Kathrin Rousk, [kathrin.rousk@bio.ku.dk](mailto:kathrin.rousk@bio.ku.dk)

**S1** **Fig** Total dissolved N (TDN), NO_3_^-^ -N and NH_4_^+^-N (mg/l) in moss leachates over the course of the experiment. Given are means ±SE (n=5).
