# Supplementary material for: The Sensitivity of Moss-Associated Nitrogen Fixation towards Repeated Nitrogen Input
Source: PLoS One. 2016 Jan 5;11(1):e0146655. doi: 10.1371/journal.pone.0146655 (PMC4712137; doi:10.1371/journal.pone.0146655)
Supplement: S2 Fig — Shown are all data points measured throughout the experiment across all N addition treatments. (DOCX) [file pone.0146655.s003.docx]

**Supporting Information**

**Manuscript title: The sensitivity of moss-associated nitrogen fixation towards repeated nitrogen input**

**Authors:** Kathrin Rousk^1,2,*^, Anders Michelsen^1,2^

^1^Department of Biology, Terrestrial Ecology Section, University of Copenhagen, Universitetsparken 15, DK-2100, Copenhagen, Denmark. ^2^Center for Permafrost (CENPERM), University of Copenhagen, Øster Voldgade 10, DK-1350 Copenhagen, Denmark. *Corresponding author: Kathrin Rousk, [kathrin.rousk@bio.ku.dk](mailto:kathrin.rousk@bio.ku.dk)

**S2** **Fig** Acetylene reduction (µmol m^-2^ d^-1^) in relation to **a.** total dissolved N (mg l^-1^), **b.** nitrate-N (mg l^-1^), **c.** ammonium-N (mg l^-1^) and **d.** the recovered inorganic N (%) in the leachate samples throughout the experiment for all treatments.
